# Supplementary material for: Aetiology and impact of bacterial bloodstream infections in mechanically ventilated COVID-19 patients: A prospective Swedish multicenter cohort study
Source: PLoS One. 2026 Jan 6;21(1):e0340476. doi: 10.1371/journal.pone.0340476 (PMC12774336; doi:10.1371/journal.pone.0340476)
Supplement: S1 Table — (DOCX) [file pone.0340476.s001.docx]

| **Significant pathogens** |
| --- |
| *Aerococcus urinae* |
| *Anaerococcus species* |
| *Bacteroides thetaiotaomicron* |
| *Bacteroides vulgatus* |
| *Citrobacter koseri* |
| *Clostridium innocuum* |
| *Enterobacter cloacae complex* |
| *Enterococcus avium* |
| *Enterococcus faecalis* |
| *Enterococcus faecium* |
| *Escherichia coli* |
| *Fusobacterium nucleatum* |
| *Klebsiella aerogenes* |
| *Klebsiella pneumoniae* |
| *Neisseria species* |
| *Parabacteroides species* |
| *Pseudomonas aeruginosa* |
| *Serratia marcescens* |
| *Staphylococcus aureus* |
| *Streptococcus pneumoniae* |
| *Veillonella species* |
| *Gemella morbillorum* |
| *Parvimonas micra* |
| *Prevotella species* |
|  |
| **Pathogens considered as possible contamination (requiring growth in at least two bottles drawn at the same event to be considered a significant patogens)** |
| *Bacillus species* |
| *Coagulase-negative staphylococci* |
| *Corynebacterium species* |
| *Corynebacterium striatum* |
| *Cutibacterium acnes* |
| *Cutibacterium species* |
| Gram-positive cocci |
| Gram-positive cocci, probably staphlococci |
| *Lactobacillus species* |
| *Staphylococcus auricularis* |
| *Staphylococcus capitis* |
| *Staphylococcus epidermidis* |
| *Staphylococcus hominis* |
| *Streptococcus anginosus* |
| *Streptococcus bovis group* |
| *Streptococcus mitis complex* |
