# Supplementary material for: Aetiology and impact of bacterial bloodstream infections in mechanically ventilated COVID-19 patients: A prospective Swedish multicenter cohort study
Source: PLoS One. 2026 Jan 6;21(1):e0340476. doi: 10.1371/journal.pone.0340476 (PMC12774336; doi:10.1371/journal.pone.0340476)
Supplement: S2 Table — Data presented as numbers (percentages). Abbreviations: Percentages: (%), ARDS: Acute respiratory distress syndrome, BMI: Body mass index, BSI: Bacterial bloodstream infection, CCI: Charlson Comorbidity Index, CRP: C-reactive protein, ICU: Intensive care unit, IL-6: Interleukin-6, IMV: Invasive mechanical ventilation, SAPS 3: Simplified acute physiology score 3, SOFA: Sequential organ failure assessment, IQR: Interquartile range, PaO2/FiO2: Partial pressure of arterial oxygen to fraction of inspired oxygen, SAPS 3: Simplified acute physiology score 3, SOFA: Sequential organ failure assessment. (DOCX) [file pone.0340476.s002.docx]

| **Variable** | **Missing data (%)** |
| --- | --- |
| **Age** | 0 (0%) |
| **Male** | 0 (0%) |
| **BMI** | 9 (2.5%) |
| **Clinical frailty scale** | 3 (0.8%) |
| **Hypertension** | 4 (1.1%) |
| **Ever smoker** | 0 (0%) |
| **Charlson Comorbidity index (CCI)** |  |
| CCI score | 0 (0%) |
| Chronic pulmonary disease | 0 (0%) |
| Rheumatologic disease | 0 (0%) |
| Liver disease | 0 (0%) |
| Diabetes | 0 (0%) |
| Diabetes with signs of organ complications | 0 (0%) |
| Malignancy | 0 (0%) |
| Moderate to severe renal disease | 0 (0%) |
| **Medication prior to admission to hospital** |  |
| Chronic steroid therapy | 0 (0%) |
| Other immunosuppresive agents | 0 (0%) |
| **PRE ICU ADMISSION RISK FACTORS** |  |
| **Community-acquired BSI** | 0 (0%) |
| **Hospital ward-acquired BSI** | 0 (0%) |
| **Duration of symptoms prior to ICU admission** | 12 (3.4%) |
| **Antibiotic treatment at hospital admission** | 0 (0%) |
| **Laboratory testing at ICU admission** |  |
| Creatinine | 9 (2.5%) |
| Leukocytes | 11 (3.1%) |
| Lymphocytes | 63 (17.8%) |
| Neutrophils | 61 (17.2%) |
| Thrombocytes | 12 (3.4%) |
| C-reactive protein (CRP) | 12 (3.4%) |
| Procalcitonin | 37 (10.5%) |
| Lactate | 8 (2.3%) |
| Bilirubin | 14 (4.0%) |
| D-dimer | 25 (7.1%) |
| Ferritin | 35 (9.9%) |
| Interleukin-6 (IL-6) | 80 (22.6%) |
| **SOFA score at ICU admission** | 40 (11.3%) |
| **SAPS 3** | 10 (2.8%) |
| **Minimum PaO2/FiO2 ratio at day 2 of intubation** | 9 (2.5%) |
| **Severity of ARDS at day 2 of intubation** | 9 (2.5%) |
| **OUTCOMES** |  |
| IMV | 1 (0.3%) |
| Length of ICU stay | 1 (0.3%) |
| Length of hospital stay | 10 (2.8%) |
| ICU mortality | 0 (0%) |
| Hospital mortality | 0 (0%) |
| 365-day mortality | 0 (0%) |
