# Supplementary material for: Aetiology and impact of bacterial bloodstream infections in mechanically ventilated COVID-19 patients: A prospective Swedish multicenter cohort study
Source: PLoS One. 2026 Jan 6;21(1):e0340476. doi: 10.1371/journal.pone.0340476 (PMC12774336; doi:10.1371/journal.pone.0340476)
Supplement: S3 Table — Abbreviations: aOR: Adjusted Odds Ratio, BSI: Bacterial bloodstream infection, BMI: Body mass index, CI: Confidence Interval, ICU: Intensive care unit. (DOCX) [file pone.0340476.s003.docx]

| **Data set used: Original data set with missing variables.** |  |  |  |
| --- | --- | --- | --- |
| **Dependent variable:** Acquisition of an ICU-acquired BSI. |  |  |  |
| **Independent variables:** | **Adjusted Odds Ratio (aOR)** | **95% Confidence Interval (CI)** | ***P-value*** |
| BMI | 1.050 | 1.002 - 1-100 | *0.040* |
| Diabetes with signs of organ complications | 2.407 | 1.172 - 4.945 | *0.017* |
| Duration of symptoms prior to ICU admission | 1.046 | 1.012 - 1.081 | *0.007* |
| **Hosmer-Lemshow test for goodness of fit:** *P = 0.84* |  |  |  |
| **Data set used: Imputated data set.** |  |  |  |
| **Dependent variable:** Acquisition of an ICU-acquired BSI. |  |  |  |
| **Independent variables:** | **Adjusted Odds Ratio (aOR)** | **95% Confidence Interval (CI)** | ***P-value*** |
| BMI | 1.058 | 1.012 - 1.107 | *0.014* |
| Diabetes with signs of organ complications | 2.657 | 1.334 - 5.292 | *0.005* |
| Duration of symptoms prior to ICU admission | 1.041 | 1.010 - 1.072 | *0.008* |
| **Hosmer-Lemshow test for goodness of fit:** *P = 0.94* |  |  |  |
