# Supplementary material for: Aetiology and impact of bacterial bloodstream infections in mechanically ventilated COVID-19 patients: A prospective Swedish multicenter cohort study
Source: PLoS One. 2026 Jan 6;21(1):e0340476. doi: 10.1371/journal.pone.0340476 (PMC12774336; doi:10.1371/journal.pone.0340476)
Supplement: S4 Table — Supplementary Table 4 shows the univariable logistic regression result, including acquiring an ICU-acquired BSI and variables known at ICU admission and their association with 365-day mortality. Data are presented as p-value, Odds Ratio and 95% Confidence Interval. P-values < 0.05 were considered significant. Covariates included in the multivariable logistic regression were age, BMI, CCI, CFS, duration of symptoms before ICU admission, history of smoking (ever smoker), ICU-acquired BSI, medication with immunosuppressive agents before hospitalisation, PaO2/FiO2 ratio at day 2 of intubation and SAPS 3. Abbreviations: aOR: Adjusted Odds Ratio, AUROC: Area Under the Receiver Operating Characteristics BMI: Body mass index, BSI: Bloodstream infection, CCI: Charlson Comorbidity Index, CI: Confidence Interval, ICU: Intensive care unit, OR: Odds Ratio, PaO2/FiO2: Partial pressure of arterial oxygen to fraction of inspired oxygen, SAPS 3: Simplified Acute Physiology Score 3 and SOFA: Sequential Organ Failure Assessment. (DOCX) [file pone.0340476.s004.docx]

| **Univariable logistic regression** |  |  |  |
| --- | --- | --- | --- |
| Dependent variable: 365-day mortality rate |  |  |  |
|  | **Odds Ratio (OR)** | **95% Confidence Interval (CI)** | ***P-value*** |
| ICU-acquired BSI | 2.111 | 1.188 - 3.749 | ***0.011*** |
| Age | 1.096 | 1.069 - 1.125 | ***<0.001*** |
| Male | 1.131 | 0.705 - 1.814 | *0.611* |
| BMI | 0.943 | 0.910 - 0.978 | ***0.001*** |
| Clinical frailty scale | 1.455 | 1.184 - 1.789 | ***<0.001*** |
| Hypertension | 1.240 | 0.812 - 1.893 | *0.320* |
| Ever smoker | 2.145 | 1.397 - 3.294 | ***<0.001*** |
| Charlson Comorbidity index (CCI) score | 1.853 | 1.559 - 2.202 | ***<0.001*** |
| Medication prior to hospital admission |  |  |  |
| Other immunosuppressive agents | 1.650 | 0.794 - 3.433 | *0.180* |
| Duration of symptoms prior to ICU admission | 1.051 | 1.018 - 1.085 | ***0.002*** |
| Antibiotic treatment at hospital admission | 0.959 | 0.631 - 1.459 | *0.846* |
| SOFA score at ICU admission | 1.014 | 0.962 - 1.068 | *0.614* |
| SAPS 3 | 1.055 | 1.035 - 1.076 | ***<0.001*** |
| PaO_2_/FiO_2_ ratio at day 2 of intubation | 0.917 | 0.882 - 0.954 | ***<0.001*** |
|  |  |  |  |
| **Multivariable logistic regression** |  |  |  |
| Dependent variable: 365-day mortality rate |  |  |  |
| **Final adjusted model** | **Adjusted Odds Ratio (aOR)** | **95% Confidence Interval (CI)** | ***P-value*** |
| ICU-acquired BSI | 3.205 | 1.611 - 6.376 | ***<0.001*** |
| Age (Years) | 1.056 | 1.022 - 1.091 | ***<0.001*** |
| BMI (kg/m^2^) | 0.950 | 0.905 - 0.996 | ***0.034*** |
| Clinical frailty scale (score) | 1.428 | 1.089 - 1.873 | ***0.010*** |
| Charlson Comorbidity index (CCI) score | 1.337 | 1.074 - 1.664 | ***0.009*** |
| SAPS 3 | 1.031 | 1.008 - 1.055 | ***0.008*** |
| PaO_2_/FiO_2_ ratio at day 2 of intubation (kPa) | 0.898 | 0.854 - 0.943 | ***<0.001*** |
|  |  |  |  |
| AUROC: 0.83 (95% CI 0.79 - 0.87) |  |  |  |
| Hosmer-Lemeshow test for goodness of fit: *P = 0.939* |  |  |  |
