# Supplementary material for: Aetiology and impact of bacterial bloodstream infections in mechanically ventilated COVID-19 patients: A prospective Swedish multicenter cohort study
Source: PLoS One. 2026 Jan 6;21(1):e0340476. doi: 10.1371/journal.pone.0340476 (PMC12774336; doi:10.1371/journal.pone.0340476)
Supplement: Attachment — Submitted filename: Response to Reviewers.docx [file pone.0340476.s006.docx]

**Response to Reviewers: PLOS ONE PONE-D-25-02714**

We want to thank the editor and reviewers for their wise and insightful comments. We have tried our very best to make adequate changes to the manuscript and we have uploaded a new improved version of the manuscript as well as a copy of the manuscript with trackable highlighted changes. We believe that your comments have made it possible for us to substantially improve the quality of the manuscript.

**Editor comments:**

**Please ensure that your manuscript meets PLOS ONE's style requirements, including those for file naming.**

*Response: We have reviewed the manuscript and the PLOS ONE’s style requirements, including those for file naming, and adjusted all the discrepancies from the requirements stated in the PLOS ONE style templates.*

**In the online submission form, you indicated that data collected for this study, including individual participant data, are not publicly available because they contain potentially identifying and sensitive patient information regulated by Swedish law. Data can be made available upon reasonable request from investigators.**

**All PLOS journals now require all data underlying the findings described in their manuscript to be freely available to other researchers, either 1. In a public repository, 2. Within the manuscript itself, or 3. Uploaded as supplementary information.**

**This policy applies to all data except where public deposition would breach compliance with the protocol approved by your research ethics board. If your data cannot be made publicly available for ethical or legal reasons (e.g., public availability would compromise patient privacy), please explain your reasons on resubmission and your exemption request will be escalated for approval.**

*Response: Dear editor, we have now tried to adjust our submission in line with your desire and the stated requirements of the journal, without breaching the regulations stated in the ethics approval and Swedish law. Furthermore, we have updated the data availability section within the submission form with further details regarding data availability.*

**Comments from reviewer #1 to the author**

**During a one-year study period at the beginning of Covid-19 pandemic, Olsson et al. conducted a prospective cohort study among mechanically ventilated Covid-19 patients in six ICUs on four study sites in Sweden. The aims of the study were to describe patient characteristics and the microbiological spectrum in blood cultures, to investigate the impact of ICU-acquired BSI on various outcomes and to explore predictors for ICU-acquired BSI on admission to ICU. Several published studies have already investigated these objectives in various settings. It is not clear from the objectives what the authors hope to learn from the study that is not already known.**

**In their study, the authors focus exclusively on patient-specific risk factors for BSI acquisition such as BMI, diabetes etc. They do not explore external risk factors such as low staff adherence to basic infection control measures (e.g. hand hygiene, especially before clean/aseptic procedures) which has been shown to influence BSI rates (e.g. see Hansen Carter et al., Infect Control Hosp Epidemiol. 2016). In many hospitals hand hygiene compliance has decreased during the pandemic in favour of wearing personal protective equipment including gloves, with nosocomial infections including central-line associated BSI being a consequence (e.g. see Schlosser et al., BMC Infect Dis 2024).**

**A prospective multi-center cohort study could have tried to address these issues, at least by exploring and reporting differences between study sites or units in the statistical analyses. If differences between study sites can be observed, what are potential explanations? E.g. did the use of alcohol-based hand rubs or of non-sterile gloves differ? Were hand sanitizers available or was there a shortage during the study period? Was there a different patient to health-care worker ratio (understaffing)?**

*Response: We want to thank reviewer #1 for the thorough review and insightful comments making it possible for us to enhance the manuscript. It is correct that there have been other studies investigating ICU acquired BSIs among patients with COVID-19. However, studies investigating this topic in the Nordic context where this study was performed is scarce, and differences in for example antimicrobial resistance and empiric antibiotic use in the Nordic countries compared to other regions have been reported (see reference #21). Hence, we believe that there might be relevant differences to uncover and that there is a knowledge gap which justifies our study. Furthermore, we think that our findings from this relatively large prospective multicenter cohort study including 354 mechanically ventilated patients adds another valuable piece to the complex puzzle of understanding ICU acquired BSIs. We also believe that our research is in line with the publication criteria of PLOS ONE: ”We evaluate research on the basis of scientific validity, strong methodology, and high ethical standards—not perceived significance”. However, we agree with the reviewer that the aim of this study could have been presented in a better way in the manuscript. We have now specified the aim in the manuscript and highlighted our objective of investigating ICU acquired BSIs among critically ill COVID-19 patients in a Nordic context. We have tried to make sure that the aim is clear and adequately presented for the reader.*

*It is correct that this study focuses on patient-specific risk factors. We have now specified this in the aims. We agree with the reviewer that investigating external risk factors such as low staff adherence to basic infection control measures (e.g. hand hygiene, especially before clean/aseptic procedures) would have been interesting. Analysing non-patient-specific risk factors was beyond the scope of our study. However, we have added a section within the discussion, highlighting that it would be interesting to investigate non-patient-specific risk factors and their associations with ICU acquired BSI in future studies.*

*In line with your suggestion, we analysed whether ICU strain/ICU burden was associated with the rate of ICU acquired BSI, we analysed whether ICU strain/ICU burden did affect the rate of ICU acquired BSIs. A univariable analysis did not show a significant association between ICU strain and the risk of acquiring an ICU acquired BSI (p = 0.155). Further, we have specified in the methods section that this study focuses on patient-specific risk factors. We do not consider ICU strain as a patient-specific risk factor. However, to make our research transparent and to answer your question properly we performed the multivariable logistic regression once again, now with ICU strain/ICU burden included in the analysis. The result of the multivariable logistic regression remained intact and ICU strain/ICU burden was not a significant predictor of ICU acquired BSI.*

*We have thoroughly discussed your response regarding site specific data. The participating hospitals were located in the same healthcare region. Hence, they were obliged to follow the same regional recommendations and had equal conditions regarding access to resources like alcohol-based hand disinfection, mouth guards and gloves. Further, they were located less than a maximum of two driving hours from each other. We have added this information to the methods section. We proceeded by further analysing the collected data. About 47% of the included patients were registered at more than one specific intensive care unit, implying that they had been transferred between different ICUs within the region. We therefore created a specific binary variable based upon whether a patient had been transferred between different ICUs or not. However, we could not show that being transferred at least once during the ICU stay significantly increased the risk of acquiring a BSI (p > 0.05). We have now added this variable to table 1*

**In general, the methods section is very thin, some of the methods only appear later in the results section or remain unclar, see below.**

*Response: This manuscript presents the results of a sub-study that was part of the prospective multicenter cohort study SWECRIT COVID-19. As the methodology regarding data collection has previously been extensively described by Didriksson et al [Reference #22 in the manuscript] we have chosen to refer to that article to keep the manuscript as concise as possible. We have added two additional sections within the method that we realized had to be clarified after taking part of your response.* ***Page 6, lines 110-112 and page 8, lines 151-154.***

**The choice of primary and secondary outcomes chosen by the authors is somewhat uncommon. Usually, secondary outcomes should help interpret the results for the primary outcome. In contrast, in this study the secondary outcome depends on the primary outcome. To make it easier to follow, these should be two different steps in the text as well as in the tables. The classification into early and late BSI is not specified in the methods section. However, I have some reservations about this somewhat arbitrary classification, see results below.**

*Response: We have now added subheadings in the discussion section to make the connection between outcome measures, results and discussion easier to follow. We have also made changes to Table 3 in accordance with your recommendation. The classification into early and late BSI is specified in the methods section.* ***Page 7, line 142-146.***

**Lines 153-156, secondary outcome measures: It is not specified whether the duration of IMV and ICU-LOS is measured from ICU admission or from BSI onset, respectively. Same for 365-day mortality, is this from admission, from discharge, from BSI onset?**

*Response: The duration for the mentioned variables is measured from ICU admission. We have now clarified this in the methods section.*

**The duration of devices present (e.g. central venous line) should be included as a continuous variable in the regression model investigating predictors for BSI, as well as the duration of ICU stay.**

*Response: Our focus in the regression model was to explore potential patient-specific predictors known at ICU admission. Hence, we have chosen not to include these variables. To further respond to your comment, we created a “time at risk”-variable based on the duration of ICU stay before the acquisition of a BSI or not and included this into the multivariable logistic regression. Including “time at risk” in the regression did not change the final result of the analysis. Hence, the risk factors remained the same, even when adjusting for ”time at risk” and we chose not to include this in the manuscript unless you object.*

**In the introduction the authors mention that Covid-19 is associated with numerous healthcare-associated infections, not only BSI. In order to explore their influence on the long-term clinical outcomes of interest they should also be included in the regression model.**

*Response: This study focuses solely on bloodstream infections and data regarding other healthcare-associated infections were not registered.*

**Table 1: Why were patients with BSI that were not acquired at the ICU not excluded? Assuming they did receive antimicrobial treatment for their BSI that was acquired elsewhere, was their risk of acquiring another BSI really comparable to that of the other patients? The same holds true for patients receiving antibiotic treatment for other infections than BSI, but that would probably decrease your study population by too many patients.**

*Response: We investigated the occurrence of community acquired BSI and hospital ward acquired BSI as we considered it to be valuable information to see the proportion of community- and hospital ward acquired BSI in comparison to the frequency of ICU acquired BSI. Further, data regarding community- and hospital ward acquired BSI was collected to make it possible for us to distinguish blood cultures implying that the patient acquired an ICU acquired BSI from blood cultures indicating that the patient acquired a non-ICU acquired BSI. Additionally we did not know on beforehand if the acquisition of a non-ICU acquired BSI would decrease or increase the risk of obtaining an ICU acquired BSI as earlier administration of antibiotics could potentially be protective of ICU acquired BSI, but at the same time the acquisition of a non-ICU acquired BSI could potentially render the patient more susceptible for acquiring additional secondary infections such as an ICU acquired BSI. Therefore, we chose not to exclude patients acquiring a community acquired BSI or hospital-ward acquired BSI from the analyses.*

**The authors state that 98% of patients received antimicrobial therapy during their ICU stay, but what was proportion receiving therapy before onset of BSI? This should be adjusted for in the logistic regression model.**

*Response:* Of the included patients *81 % received antibiotics within the first 12 hours from ICU admission. As a BSI was considered an ICU acquired BSI if acquired > 48 hours after ICU admission, all patients that received antibiotics within the first 12 hours from ICU admission received antibiotics prior to any ICU acquired BSI was present. A Chi^2^-test did not show any relationship between receiving antibiotics within the first 12 hours from ICU admission and ICU acquired BSI (p=0,498). We unfortunately do not have continuous data on when antibiotics were administered in the data set which makes it difficult to pinpoint which patients received antibiotics prior to the acquisition of an ICU acquired BSI. It would also be difficult to interpret the data as the reason for administration of antibiotics may vary. For most cases, early antibiotic treatment was based on clinical suspicion of a bacterial co-infection given that the threshold for administering empirical antibiotic treatment for the severely ill patients included in this study was relatively low, yet some patients received targeted antibiotic treatment against verified infections outside the bloodstream. Further, which antibiotics that were administered might impact the data as well. Hence, based upon the statistical analysis presented above, in combination with the clinical reasoning, we have kept the current logistic regression model intact. However, we understand the thought process behind the reviewer’s comment and acknowledge that this might be an interesting variable to focus on in future studies. After careful consideration of the reviewer’s comment, we have also added this to the limitations section.*

**Lines 247-251: The classification of ICU acquired BSI into early and late onset with a cutoff of 7 days seems arbitrary to me. Rather than exploring it as an outcome, the authors should take advantage of this continuous variable and adjust the analysis of the other risk factors for the length of ICU stay prior to BSI onset, see above. I would assume that the risk of acquiring BSI increases with duration of ICU treatment, i.e. time at risk.**

*Response: The chosen method regarding dividing ICU acquired BSI into early and late BSI was chosen with careful consideration. We used a method previously described and used by Tabah et al. in an international multicenter study published in the high impact journal Intensive Care Medicine [Reference #25 in the manuscript]. The chosen method of dichotomizing the ICU acquired BSI into early and late rendered it possible to in a comprehensible and reader friendly way present important differences regarding early and late ICU acquired BSI which potentially might have been compromised if analysing it as a continuous variable. Further, handling the variable as a continuous variable would potentially cause problems due to the skewed data. We understand that in some aspects there might have been potential benefits of analysing the data as a continuous variable, but we hope that our final decision of analysing the data as a dichotomized variable based upon the method presented by Tabah et al. is understandable and reasonable.*

**Table 3: Mentioning outcomes measured at the end of the hospital stay/one year after hospital discharge in the same table as the risk factors is difficult to grasp for the reader.**

*Response: We have made changes to the table in accordance with the reviewer’s response. See the updated Table 3. We have made a similar change to Table 1. Please, see the updated Table 1.*

**Figure 2: The Y-Axis title is missing.**

*Response: The figure has now been corrected. See updated Figure 2.*

**Lines 315-318: The authors discuss devices as points of entry of bacteria. However, neither do they explore the presence of these devices and their handling as risk factors for BSI, nor do they discuss the preventability of these infections.**

*Response: As this study focuses on severely ill patients with COVID-19 requiring invasive mechanical ventilation the number of patients that did not have a central venous catheter and/or peripheral venous catheters during most of their ICU stay is arguable very few, if any. The handling of these devices was not investigated as this falls out of the scope of this study. We have added this to the limitations to highlight that in future studies, data regarding peripheral and central venous catheters should be considered to eventually collect and analyse.*

**Lines 322-325: The conclusion is correct that from the results at hand the authors cannot distinguish between chicken and egg. As suggested above they should at least differentiate between exposure and outcome time, i.e. time before and after developing BSI.**

*Response: All of our patient specific factors were present before the ICU acquired BSI. Please read the response stated above regarding time at risk.*

**Lines 327-334: The authors indirectly state that their statistical model did not seem to include the relevant predictors for BSI. For improvements to the model see comments above.**

*Response: Please read the responses stated above.*

**Comments from Reviewer #2 to the author**

**Reviewer #2: The authors refers 98% of the study subjects received antibiotic therapy at some point their ICU stay. They do not mention the spectrum of this antibiotics.**

*Response: We thank the reviewer #1 for the thorough review and insightful comments making it possible for us to enhance the manuscript. In general, the first line of antibiotic treatment used in our region was third generation Cephalosporins (mainly Cefotaxime), and the second line of treatment was Carbapenems and Piperacillin-tazobactam (penicillin combined with a beta-lactamase inhibitor). We have added this to the manuscript.* ***Page 8, lines 151-154.***

**There is no definition of antibiotic resistance i.e Gram-negative bacteria as Enterobacteriaceae with extended-spectrum β-lactamase production or piperacillin-tazobactam resistance, and Acinetobacter spp and Pseudomonas aeruginosa with piperacillin-tazobactam or carbapenem resistance.**

*Response: The situation regarding antimicrobial resistance in the Nordic countries has been reported to not be as severe as for example in the eastern and southern parts of Europe (see reference #21 in the manuscript). Hence, we consider it important to highlight the Nordic aspect of the study. Further, the low resistance decreases the need of specifying antibiotic resistance for specific species occurring in the study. Additionally, the rate of deaths associated with antimicrobial resistance is lower in the Nordic countries compared to most other countries according to a Lancet article published in 2024 (reference #36 in the manuscript). We acknowledge the insightfulness of the reviewer’s comment; hence we have added information about the lower rate of antimicrobial resistance in the Nordic countries into the background section* ***(Page 5, lines 91-95)*** *as well as into the discussion* ***(Page 20, lines 321-331)****.*

**In this study Staphylococcus aureus was the most common pathogen identified, usually in this ICU context there is a swab examination. Why did they not collect swabs to evaluate the presence of methicillin-resistant Staphylococcus aureus (MRSA) or vancomycin-resistant Enterococcus (VRE)?**

*Response: In the Skåne Region, where the study was conducted, there is no routine of performing a general swab examination of all patients to identify resistant pathogens like MRSA and VRE, except for patient’s transferring in from ICU’s outside of the Nordics. During the study period, transfer of critically ill patients from abroad was rare. Furthermore, see the previous response about the situation regarding antimicrobial resistance in the Nordic countries.*

**The most frequent pathogens reported in ICU, particularly when the aim of the study is to investigate the bacterial aetiology. The results shows 45% of the subjects developed late ICU acquired BSI, they received antibiotics at the hospital admission, it is interesting to know about any antibiotic rotation strategies used during the hospitalization, and the therapy the subjects received previous the hospitalization, with the intention to evaluate pre-ICU admission risk factors.**

*Response: We unfortunately do not have any information on antibiotic therapy prior to hospital admission. In general, as previously mentioned in the response letter, the first line of antibiotic treatment used in our region was third generation Cephalosporins (mainly Cefotaxime), and the second line of treatment was Carbapenems and Piperacillin-tazobactam. As already mentioned, we have added this to the manuscript.* ***Page 8, lines 151-154****.*
