# Supplementary material for: Aetiology and impact of bacterial bloodstream infections in mechanically ventilated COVID-19 patients: A prospective Swedish multicenter cohort study
Source: PLoS One. 2026 Jan 6;21(1):e0340476. doi: 10.1371/journal.pone.0340476 (PMC12774336; doi:10.1371/journal.pone.0340476)
Supplement: Attachment — Submitted filename: Response_to_Reviewers_auresp_2.docx [file pone.0340476.s007.docx]

**Response to Reviewers, Second Revision: PLOS ONE PONE-D-25-02714**

We want to thank the editor and reviewers for their wise and insightful comments. We have tried our very best to make adequate changes to the manuscript and we have uploaded a new improved version of the manuscript as well as a copy of the manuscript with trackable highlighted changes. We believe that your comments have made it possible for us to substantially improve the quality of the manuscript.

**Reviewer #3:**

**The most important problem inherent to the manuscript is the outcome, as stated in the previous reviews. The pathogens to cause BSI in ICU setting is well known issue and it is not unique even in Nordic countries. The manuscript did not show many, if any, new findings other than what we already know and the Nordic setting would not make this manuscript unique still.**

*Response: There is unclarity about the incidence of BSIs as well as the microbiological spectrum in the Nordic setting. Further, no consensus exists on factors that can predict an ICU-acquired BSI on ICU admission in critically ill COVID-19 patients. An ICU-acquired BSI is associated with worse outcome, and this manuscript adds to the existing research and emphasizes the importance of active vigilance on the development of an ICU-acquired BSI.*

**Reviewer #5:**

**1. The current manuscript often reads as implying causality (“BSI increased mortality”). Because this is an observational cohort, causal claims must be tempered and rephrased as associations. The authors acknowledge this in the discussion, but the abstract and conclusion still overstate causality. More importantly, the temporal relationship between LOS/IMV duration and BSI is not rigorously handled. Patients who remain longer in ICU have more time at risk for BSI. The authors should perform time-to-event analyses such as Kaplan-Meier for time to first ICU-acquired BSI.**

*Response: This is indeed an important remark. The primary aim of this study was to describe the aetiology and frequency of BSI. Secondary aims included associations between BSI and duration of IMV and LOS as well as all-cause mortality. We have clearly written throughout the manuscript, including the abstract, that the secondary objectives are mere associations and have not implied any causality from our findings. In line with reviewer 5’s remark, we have further toned down the association with mortality (****Page 2, lines 55-56; page 18, lines 299-300; page 22, lines 402-404****). We believe that further analyses, such as time-to-event analyses for time to first ICU-acquired BSI, would not bring us any closer to the possible causal relationship between BSI and mortality. This has been further clarified in the discussion (****Page 20, lines 349-354****).*

**2. Blood cultures were drawn based on clinical judgement. What were the typical clinical indications? Was there a standardized sampling protocol across sites? This is necessary because differential testing can bias apparent incidence.**

*Response: We have added additional information on regional guidelines for blood sampling in patients with a suspected severe infection or sepsis. The participating hospitals follow the guidelines from Surviving Sepsis Campaign Guidelines (****Page 7, lines 135-143****).*

**3. Provide a clear table listing all pathogens, counts, and percent of total BSIs.**

*Response: Please see supplementary table 1 for information regarding pathogens that were considered significant if they were identified in a blood culture and figure 2 for a presentation of how frequently a specific bacterial species was identified.*

**4. Provide a table summarising empiric agents used (first-line and second-line), durations, and the proportion who received antibiotics prior to the positive blood culture.**

*Response: Unfortunately, that specific data is not available in more detail than what is already presented.*

**5. Provide antimicrobial susceptibility patterns (resistance to 3rd-generation cephalosporins, MRSA, VRE, ESBL, carbapenem resistance) for the isolates. This affects interpretation of empiric therapy appropriateness and outcome.**

*Response: The situation regarding antimicrobial resistance in the Nordic countries has been reported to not be as severe as for example in the eastern and southern parts of Europe (see reference #21 in the manuscript). Hence, we consider it important to highlight the Nordic aspect of the study. Further, the low resistance decreases the need of specifying antibiotic resistance for specific species occurring in the study. Additionally, the rate of deaths associated with antimicrobial resistance is lower in the Nordic countries compared to most other countries according to a Lancet article published in 2024 (reference #36 in the manuscript). We acknowledge the insightfulness of the reviewer’s comment and since the first submission we have added information about the lower rate of antimicrobial resistance in the Nordic countries into the background section* ***(Page 5, lines 90-95)*** *as well as into the discussion* ***(Page 20, lines 321-331)****.*

*In addition, we have checked the aggregated data from the intensive care register. There was a growth of multiresistent bacteria (MRSA, ESBL or VRE) in 0,9 % of the ICU admissions suffering from COVID-19 at the participating ICUs.*

**6. BSIs in ICU are strongly associated with intravascular devices. The current dataset lacks device-level exposure variables (presence/duration of central venous catheters, number of line days, insertion practices, catheter care bundles), a limitation the authors note, but these data are critical. If such data are available in the records, they should be added and analyzed (CLABSI rates). If not available, the authors should, explicitly state this limitation(already partly done) and avoid implying modifiable device factors without data.**

*Response: We have adjusted the manuscript in accordance with this response by adjusting the included tables and the manuscript.*

**7. The multivariable model aiming to predict BSI at ICU admission has AUROC 0.66, which is poor. Authors should report how multicollinearity was assessed and the full list of variables considered (you mention p<0.2 rule but please provide a table of candidate variables).**

*Response: Variables with a p<0.2 in the non-parametric analysis (see table 1) were included in the logistic regression. All variables with a p<0.2 in the non-parametric tests were analysed in univariable logistic regressions. Variables with a p<0,2 in the univariable logistic regression were included in the multivariable logistic regression. Please, see table 2 for data regarding the logistic regressions.*

*We have assessed multicollinearity by testing for strong correlations with Spearmans- and Point-Biserial-test and we could not identify any strong correlations (all correlations below 0,3) between any variables included in the multivariable regression analysis. We have now added this to the manuscript (****Page 9-10, lines 188-191****).*
